# EHMTI-0128. Headache as an initial symptom with the patients treated at the intensive care unit of the Clinic of Neurology 1997 – 2013

**DOI:** 10.1186/1129-2377-15-S1-D75

**Published:** 2014-09-18

**Authors:** N Vukasinovic, S Jolic, V Milosevic, M Zivkovic, P Slankamenac

**Affiliations:** 1 Dept.of Neurology, Clinical center Nis, Nis, Serbia; 2 Dept.of Neurology, Clinical center Vojvodina, Novi Sad, Serbia

## Introduction

At the intensive care unit of the Clinic of Neurology of the Clinical centre Nis, within the period 1997 – 2013 6852 patients were treated, of which 4941 patient (72,11%) with cerebrovascular diseases. 2237 (45,2%) had headache as an initial symptom.

## Material and methods

The database of the hospitalized patients who suffered from headache as an initial symptom (verified with imaging techniques CT and MRI) of the Clinic of neurology at the Intensive care unit was analyzed.

4941 patient (2750 men s.55,7% and 2191 women s.44,3%) average age 63,11 (SD 0,117) years, 62,52 +/- 11,884 years for men 63,84 +/- 13,068 years for women. With TIA diagnose 132 (4,8%) men and 104 (4,7%) women were treated with headache 29 (12,2%), with subarachnoid haemorrhage 163 (5,9%) men and 257 (11,7%) women, with headache 346 (82,3%), with the diagnose of intracerebral hemorrhage 758 (27,6%) men and 561 (25,6%) women, with headache 846 (64,2%). Diagnosed with ischaemic brain stroke 1360 (49,5%) men and 978 (44,6%) women, with headache 893 (38,2%), with the diagnose of undefined brain stroke 196 (7,1%) men and 160 (7,3%) women, with headache 123 (34,7%) and with the diagnose of sequel conditions after the brain stroke 141 (5,1%) men and 131 (6,0%) women, without headache.

## Conclusion

There is no significant difference of the presence of headaches as initial symptom according to sex. There is a frequency of headaches with ischaemic brain stroke (893 patients, that is 39,9% of all headaches) contrary to the expectations.

